# Starch Digestion Enhances Bioaccessibility of Anti-Inflammatory Polyphenols from Borlotti Beans (*Phaseolus vulgaris*)

**DOI:** 10.3390/nu12020295

**Published:** 2020-01-22

**Authors:** Lucia Margarita Perez-Hernandez, Kartika Nugraheni, Meryem Benohoud, Wen Sun, Alan Javier Hernández-Álvarez, Michael R. A. Morgan, Christine Boesch, Caroline Orfila

**Affiliations:** 1 Nutritional Sciences and Epidemiology, School of Food Science and Nutrition, University of Leeds, Leeds LS2 9JT, UK; luciam.perez88@gmail.com (L.M.P.-H.); fskn@leeds.ac.uk (K.N.); fs16ws@leeds.ac.uk (W.S.); A.J.HernandezAlvarez@leeds.ac.uk (A.J.H.-Á.); M.Morgan@food.leeds.ac.uk (M.R.A.M.); C.Bosch@leeds.ac.uk (C.B.); 2 Keracol Ltd., Nexus, Discovery Way, Leeds LS2 3AA, UK; m.benohoud@keracol.co.uk

**Keywords:** beans, domestic processing, polyphenols, bioaccessibility, starch digestion, in vitro digestion, anti-inflammatory effect, IL1β, iNOS

## Abstract

The consumption of beans has been associated with chronic disease prevention which may be attributed to the polyphenols present in the seed coat and endosperm. However, their bioaccessibility is likely to be limited by interactions with bean matrix components, including starch, protein and fibre. The aim of this project was to evaluate the effect of domestic processing and enzymatic digestion on the bioaccessibility of polyphenols from Borlotti beans (*Phaseolus vulgaris*) and to test their anti-inflammatory properties in a macrophage cell model. In vitro digestion of cooked beans released twenty times more polyphenols (40.4 ± 2.5 mg gallic acid equivalents (GAE)/g) than domestic processing (2.22 ± 0.1 mg GAE/g), with starch digestion contributing to the highest release (30.9 ± 0.75 mg GAE/g). Fluorescence microscopy visualization of isolated bean starch suggests that polyphenols are embedded within the granule structure. LC-MS analysis showed that cooked Borlotti bean contain flavonoids, flavones and hydroxycinnamic acids, and cooked bean extracts exerted moderate anti-inflammatory effects by decreasing mRNA levels of IL1β and iNOS by 25% and 40%, respectively. In conclusion, the bioaccessibility of bean polyphenols is strongly enhanced by starch digestion. These polyphenols may contribute to the health benefits associated with bean consumption.

## 1. Introduction

Common beans (*Phaseolus vulgaris*) are traditional staple foods in many countries around the world [1,2] and their consumption has been associated with health benefits in both undernourished [3] and overnourished populations [4,5]. Beans are a source of macro and micronutrients including lysine-rich proteins, low glycaemic index carbohydrates, vitamins, minerals, fibre, and phytochemicals including polyphenols [1]. Epidemiological studies suggest that a diet including beans may be protective against cancer [6,7], diabetes [8,9] and cardiovascular diseases [10,11]. In vitro and in vivo studies have shown beneficial effects of bean polyphenols, such as anti-carcinogenic, antioxidant and anti-inflammatory properties [12,13,14].

Domestic preparation of plant foods typically leads to a decrease in antioxidants including polyphenols since these compounds are relatively unstable during thermal processing [15]. Beans are generally soaked and cooked to increase the palatability and to remove anti-nutritional factors such as lectins and protease inhibitors [16]. Domestic preparation may decrease the potential health benefits of beans.

*P. vulgaris* beans display large genetic and phenotypic diversity [2]. Different cultivars have been shown to contain a wide array of polyphenols including hydroxycinnamic acids, flavonoids, condensed tannins and anthocyanins [17,18,19,20,21]. Polyphenols are not uniformly distributed in plant tissue and may be present in free or polymerized forms, as well as esterified or complexed with proteins and cell wall polysaccharides [22,23]. In addition, interactions between polyphenols and starch have also been observed using in vitro systems [24,25,26,27,28,29]. However, studies that have evaluated the release of polyphenols upon enzymatic in vitro digestion have been inconclusive. LaParra et al. (2008) found that the amount of polyphenols solubilised from white, red and black beans following in vitro digestion decreased two- to three-fold in comparison to those extracted with acidified methanol [30]. Akillioglu and Karakaya (2010) found a similar decrease when digesting common and pinto beans using pepsin and pancreatin, followed by dialysis [31]. Faller et al. (2012) found no differences between the polyphenols released by in vitro digestion and solvent extraction of feijoada (a dish containing black beans) [32]. In non-legume foods, mixed findings have also been reported. Gil-Izquierdo et al. (2002) reported five-fold decreases in most polyphenols from orange and strawberry products following digestion [33]. Bouayed et al. (2012) found that intestinal in vitro digestion of apples resulted in a considerable decrease in the total polyphenolic content after digestion [34]. Wootton-Beard et al. (2011) found that in vitro digestion enhanced the release of polyphenols, antioxidant and scavenging capacities of a range of fruit and vegetable juices compared to undigested samples, with varying effects depending on the juice [35]. Mandalari et al. (2013) found that more than 90% of the polyphenols from pistachios were released following simulated gastric digestion, with the remaining solubilized in the duodenal phase [36]. Finally, Miranda et al. (2013) found that up to five times more chlorogenic acid was released during the gastric phase compared to undigested potato samples [37]. None of these studies have investigated the effect of starch digesting enzymes independently of protease.

The aim of this study was to evaluate the in vitro bioaccessibility of Borlotti bean (*P. vulgaris*) polyphenols and to determine the contribution of protein and starch enzymatic digestion to their release from the bean matrix. In this paper, we define bioaccessibility as ‘the quantity of a specific compound that is released from the food matrix during digestion and is available to the intestinal cells for potential absorption’ [38]. Furthermore, the anti-inflammatory activity of cooked Borlotti bean extract was assessed in cultured RAW 264.7 macrophages, a cellular model of inflammation. We hypothesize that in vitro digestion will enhance the release of bean polyphenols and that these phytochemicals will modulate the expression of inflammatory markers in macrophages.

## 2. Materials and Methods

### 2.1. Bean Samples and Domestic Processing

Borlotti beans (*P. vulgaris*) were purchased from a supermarket in Leeds, UK. Beans were soaked overnight (16 h) in ultrapure water (1:5, *w*:*v*), drained and boiled for 10 min in water (1:10, *w*:*v*) followed by simmering for 1 h, as advised by the manufacturer. Aliquots of soaking and cooking water were kept at −20 °C for polyphenol analysis. Cooked beans were either subjected to digestion or freeze-dried for polyphenol extraction. Raw or freeze-dried cooked bean samples were milled to a fine powder and extracted with acidified methanol (3% trifluoroacetic acid) in a 1:20 ratio (*w*/*v*) by vortexing for 1 h. The supernatants of extractions were collected by centrifugation (9000× *g*) and kept at −20 °C for polyphenol analysis.

### 2.2. In Vitro Digestion of Starch and Protein

The in vitro digestion method used in this study was optimized to achieve the highest starch hydrolysis possible. Optimization was performed by treating the cooked bean with increasing concentrations (100–1000 units) of porcine pancreatic α-amylase at different incubation times (2, 4, 6, 8 and 16 h) in combination with a fixed amount of amyloglucosidase (80 units) and a further protease (30 U) digestion (Supplementary Material Figures S1 and S2). The optimal amount of pancreatic α-amylase was found to be 250 U/mL over 16 h, as no further increases in glucose released were observed with higher levels of enzyme. This is close to the enzyme concentration of 200 U/mL used in the simulated gastro-intestinal digestion model developed by the INFOGEST consortium [39,40]. Starch digestions were performed in a 50 mM sodium maleate buffer containing 2 mM CaCl_2_ (pH 6.0), according to McCleary [41], with some modifications [42]. Initially, cooked beans (1 g) were homogenized (40 mL buffer) and digested with different amounts of porcine pancreatic α-amylase (250 U/mL, Sigma Aldrich, Gillingham, UK) for 16 h at 37 °C. Amylase digestion was followed by digestion with 80 U/mL amyloglucosidase (from *Aspergillus niger*, Megazyme International, Bray, Ireland) for 60 min at 60 °C, pH 4.5. Samples were boiled for 5 min to stop further enzymatic activity and aliquots of the supernatants were kept at −20 °C for further analysis. For protein digestion, homogenized beans were digested with 30 U/mL protease (subtilisin A from *Bacillus licheniformis,* Megazyme International, Bray, Ireland) for 30 min at 60 °C, pH 6.0. Samples were boiled for 5 min to stop further enzymatic activity. The supernatants of digestion were collected by centrifugation and kept at −20 °C for polyphenol analysis.

### 2.3. Acid Hydrolysis of Fibre

The residues of starch and protein digestion were collected by filtration through sintered glass, washed with series of acetone, dried in an oven at 80 °C and weighed to obtain a measurement of fibre content. The fibre was hydrolysed with 1 M HCl (10 mL) at 100 °C for 1 h. The supernatant of hydrolysis was collected by centrifugation and kept at −20 °C for polyphenol analysis.

### 2.4. Determination of Total Polyphenolic Content (TPC) in Bean Extracts

TPC content was determined by the Folin–Ciocalteu assay [43] with a few modifications. Briefly, 50 µL of cooking water, soaking water, digestion or extraction supernatants, were mixed with 50 µL of Folin–Ciocalteu reagent (1 N), 150 µL of Na_2_CO_3_ (20%, *w*/*v*) and 750 µL of water. The mixture was incubated for 30 min in the darkness and the absorbance measured at 765 nm with a double beam spectrophotometer. Results were expressed as mg gallic acid equivalents (GAE) per g of dry weight.

### 2.5. Separation of Polyphenols Using Liquid Chromatography–Mass Spectrometry (LC–MS)

The LC–MS analysis was carried out on Agilent 1200 LC with a Bruker HCT Ultra Ion Trap for MS detection and a photodiode array detector (DAD) for UV/V is measurements. The mobile phase consisted of acetonitrile/water, both with 0.1% formic acid. The gradient increased from 2–95% of acetonitrile over 1 min. The column used was Phenomenex Kinetex (50 × 2.1 mm and 2.6 µm particle size). The flow rate applied for the analysis was 1.3 mL/min. Putative identification of the polyphenols was done using information available on the phenol explorer database [44] and the information published by Lopez et al. (2013) [20].

### 2.6. Starch Isolation and Microscopy

Starch isolation of beans was performed as described by Bustos et al. (2004) with a few modifications [45]. Briefly, 5 g of raw beans were homogenized in 250 mL 50 mM Tris (pH 7.5) with 10 mM EDTA and 0.5 g/L sodium metabisulfite at 4 °C and filtered through four layers of cheesecloth. The filtrate was centrifuged at 20,000× *g* and 4 °C for 15 min. The pellet was washed three times using extraction medium, then twice in acetone, and centrifuged as mentioned previously. The final pellet was dried overnight at room temperature. Isolated bean starch was fixed in water and analysed with an Olympus BH2 fluorescence microscope (Life Solutions, Tokyo, Japan). Pictures of native and gelatinized (from cooked bean) starch were taken under white and UV light.

### 2.7. Determination of Cytotoxicity and Anti-Inflammatory Activity

Murine RAW 264.7 macrophages were obtained from the European Collection of Authenticated Cell Cultures (ECACC). Macrophages were cultured in Dulbecco’s modified Eagle’s medium (DMEM), high glucose (4.5 g/L), supplemented with 10% (*v*/*v*) foetal bovine serum, and 1% antibiotics mix. Cells were grown in a humidified incubator at 37 °C and 5% CO_2_ and medium changed every second day. Methanolic extracts of cooked beans were evaporated with a Genevac EZ-2 ENVI and the resulting pellet was resuspended in 99% dimethyl sulfoxide (DMSO) to prepare a stock solution (200 mg/mL). Working solutions (50, 100, 200 μg/mL) were prepared by dissolving the stock solution in DMEM. The final DMSO concentration of the working solutions was < 0.1%.

The neutral red assay was applied to evaluate the cytotoxicity of bean extracts as previously described by Boesch-Saadatmandi et al. (2011) [46]. Briefly, cells in 24-well plates were incubated with bean extract (50–200 μg/mL) for 24 h, after which the medium was changed to DMEM containing 60 µg/mL neutral red. After 2 h, medium was removed and the dye extracted from cells using a mixture of ethanol, deionized water and glacial acid (50:49:1). The absorbance was read at 540 nm using a Tecan spark 10 M plate reader and the viability calculated in percentage of control cells. Solvent controls were included (0.1% DMSO).

Cells were incubated with increasing concentrations of bean extract (50, 100, 200 μg/mL) and sulforaphane as a positive inhibition control (5 µM) for 1 h, and then stimulated with LPS for 6 h (100 ng/mL). Cells were washed with PBS, lysed with Trisure (Bioline, London, UK) and frozen for later RNA isolation. Experiments were performed in three independent passages in duplicate.

RNA was isolated following the manufacturers’ instructions and quantified using Nanoquant plate (Tecan Spark 10 M, Männedorf, Switzerland). iScript kit (Biorad, London, UK) was used for cDNA synthesis. Real time PCR was performed using the SensiMix SYBR High-ROX Kit and the StepOne real time cycler from ThermoFisher scientific. The primers used were for the following target genes: *interleukin 6 (IL-6), interleukin 1 beta (IL1β)* and *nitric oxide synthase (iNOS)*. The housekeeper gene used was *beta actin (ACBT)*. Forward (5′-3′) and reverse (3′-5′) sequences of the target genes used in this study are listed in Table 1. Each cDNA sample was run in duplicate. Results were calculated with ΔΔ*C_T_* method and expressed as the ratio of target gene to housekeeper gene.

### 2.8. Statistical Analysis

Statistical analysis was performed using SPSS 24.0 statistical package. For digestion experiments and polyphenol determination, statistical differences between the means of three independent experiments were analyzed by one-way ANOVA, followed by multiple comparisons using the Tukey test (*p* < 0.05). Cell culture data were analyzed with one-way ANOVA followed by Dunnett’s *t*-test to compare treatments with control group. Significance was accepted at *p* < 0.05.

## 3. Results

### 3.1. Effect of Domestic Processing, Enzymatic Digestion and Fibre Hydrolysis on Polyphenol Release

**Figure 1:**
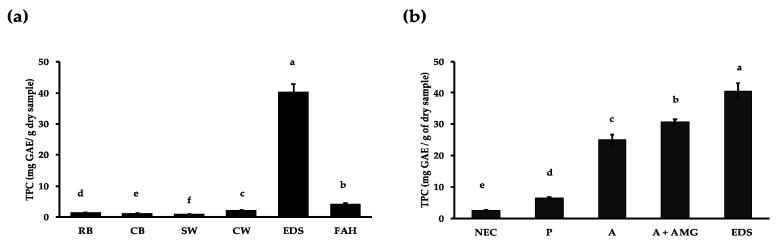
Total polyphenol content (TPC) in bean fractions (**a**) and during digestion with individual enzymes (**b**). TPC content was evaluated in raw beans (RB; acidified methanol extraction), cooked beans (CB; acidified methanol extraction), soaking (SW) and cooking water (CW), enzymatic digestion supernatants (EDS which refers to digestion with amylase, amyloglucosidase and protease) of cooked beans and fibre acid hydrolysis supernatant (FAH). (**b**) TPC in supernatants following digestion with protease (P), pancreatic α-amylase (A), pancreatic α-amylase and amyloglucosidase (A + AMG) and combined enzymes (EDS). No enzyme control (NEC) refers to undigested bean supernatant. Different letters indicate statistically different values among samples according to a post-hoc comparison (Tukey’s test) at *p* ≤ 0.05.

As shown in Figure 1a, the soaking process led to a small amount of polyphenols being released from Borlotti beans (0.9 ± 0.0 mg GAE/g sample) which is lower than the amount that was extracted from ground raw beans with acidified methanol (2.3 ± 0.7 mg GAE/g sample). Upon cooking, polyphenols were released into the cooking water (2.2 ± 0.1 mg GAE/g sample) and the extraction of cooked beans with acidified methanol extracted a further 1.3 ± 0.3 mg GAE/g dry sample. Enzymatic digestion released 20 times more polyphenol (40.42 ± 2.49 mg GAE/g sample) than domestic processing. Only a small amount of polyphenols (4.15 ± 0.45 mg GAE/g sample) was released from the fibre residue compared to in vitro digestion.

The effect of individual enzymes on polyphenol release from Borlotti beans are shown in Figure 1b. When the cooked bean sample was subjected to the in vitro digestion conditions, but without enzymes, 2.25 ± 0.16 TPC (mg GAE/g sample) were released, which is similar to the amount released during cooking. When starch and protein were digested separately, the TPC content increased to 30.78 ± 0.75 and 6.53 ± 0.46 (mg GAE/g sample), respectively. The effects of the various enzymes on polyphenol release appear to be additive, rather than synergistic.

It should be noted that the digestion method was optimized to achieve maximum starch digestion, as shown in Figure S1. The amylase concentration (250 U) is similar to the concentration used in the INFOGEST digestion protocol (200 U, [39,40]).

**Figure 2:**
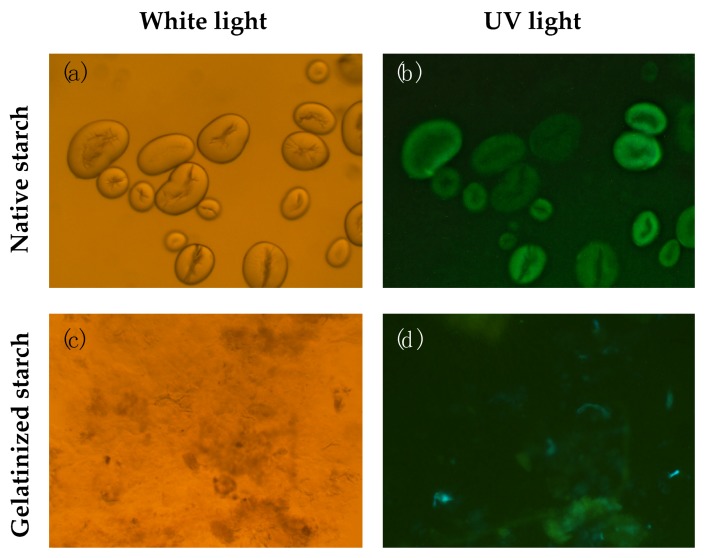
Microscopic images of native and gelatinized Borlotti bean starch with the same frame, observed under white and UV light illumination (280 nm). Starch granules isolated from Borlotti beans observed under white light illumination with phase contrast (**a**) and observed under UV light (**b**) indicating the localization of polyphenols or other fluorescent molecules within the granules. The starch granule structure is lost after gelatinization (**c**) florescence is still observed in gelatinized starch aggregates (**d**).

Using microscopy, we have observed that starch granules isolated from Borlotti beans fluoresce under UV light (Figure 2b), indicating the localization of polyphenols or other fluorescent molecules within the granules. The fluorescence is not lost upon washing with water or acetone, indicating strong interactions that are not disrupted by solvents (data not shown). When the granules were gelatinized, the granule structure was lost (Figure 2c), but some fluorescence was observed in the gelatinized starch aggregates (Figure 2d).

### 3.2. Polyphenol Profile of Cooked Bean Extract

LC–MS analysis of the cooked bean extract revealed the presence of a mixture of polyphenols including hydroxycinnamic acids (chlorogenic acid), flavonoids (procyanidin B and petunidin derivatives) and flavones (apigenin derivatives) (Table 2, Figure S3). A number of the proposed polyphenol derivatives do not have readily available standards, and therefore the mass of the ions could not be used to confirm parent compounds. We found this to be a real challenge in the field, where researchers assume the parent compound from a fragmented ion. We have refrained from making assumptions by only indicating the potential parent structure. Until reference standards are available for derivatives, such as the glycosides and acetylated compounds, it will not be technically possible to confirm the composition. The chromatogram does suggest that bean extract contains a mixture of polyphenols. This extract was used to study the anti-inflammatory effects of bean extract in macrophages.

### 3.3. Anti-Inflammatory Properties of Cooked Bean Extracts in RAW 264.7 Macrophages

**Figure 3:**
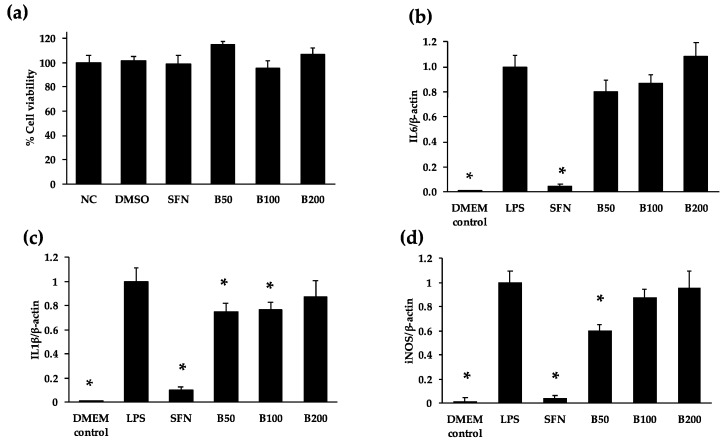
Effect of Borlotti bean extract on (**a**) cell viability and (**b**–**d**) pro-inflammatory cytokine expression in macrophages. (**a**) Murine RAW264.7 macrophages were incubated with 50, 100 and 200 μg/mL as well as 5 μM sulforaphane (SFN) and DMSO (1%) and cell viability was assessed by neutral red assay. Negative control (NC) refers to DMEM medium only. (**b**) Effect of Borlotti bean extracts on mRNA inflammatory target genes *IL6* (**b**), *IL1-β* (**c**) and *iNOS* (**d**). Macrophages were incubated with 50 (B50), 100 (B100) and 200 μg/mL (B200) cooked Borlotti bean extract and stimulated with LPS (100 ng/mL). Controls used were unstimulated medium control (DMEM), LPS-stimulated control (LPS) and sulforaphane (5 μM) control (SFN). * indicates statistically different values among samples according to a post-hoc comparison (Dunnett’s test) at *p* < 0.05.

When incubated with concentrations up to 200 μg/mL of cooked bean extract, macrophage viability was not affected (Figure 3a). The exposure of cells with cooked bean extract at concentrations of 50 and 100 μg/mL decreased mRNA levels of inflammatory marker IL1β by 25% and 22% respectively, in comparison to the LPS control (Figure 3c). In addition, iNOS mRNA levels were decreased by 40% at a concentration of 50 μg/mL, but not at higher concentrations (Figure 3d). No significant change in IL6 mRNA levels was observed (Figure 3b). The effect of bean extract was significantly smaller than the effect of sulforaphane (5 µM) for all three target genes.

## 4. Discussion

Relatively small amounts of polyphenols were solubilised during soaking and cooking; these polyphenols are likely to originate from the coloured seed coat and may have functions in protecting seeds from environmental stress and pathogen attack. Our TPC values of whole Borlotti beans are comparable to other *P. vulgaris* polyphenol studies [18]. Around two times more polyphenols were released upon cooking than soaking. Xu and Chang (2008) observed similar effects during soaking and cooking of *P. vulgaris* beans [19]. Soaking water is often discarded and not consumed, and is therefore unlikely to contribute to health effects. Beans are generally soaked before cooking in order to remove anti-nutritional factors such as lectins and protease inhibitors [16]. Polyphenols are solubilized into the water during the soaking process, resulting in the decrease in TPC and the loss of their potential health benefits. Domestic preparation of food of vegetable origin normally leads to a decrease in antioxidants, including polyphenols, since these compounds are relatively unstable to thermal processing [15]. Satwadhar et al. (1981) studied the effect of cooking on the polyphenol content of moth bean (*Vigna aconitifolia*) [45]. In the mentioned study, cooking was performed by boiling (100 °C) for 10, 15 and 80 min. It was observed that the polyphenol content decreased by 50% after 10 min of boiling. No further loss of polyphenols was observed after 15 and 80 min of boiling. In this study, the soaking and cooking times were those provided by the manufacturer. We decided to follow the processing methodology suggested by the manufacturer to keep TPC values relevant to the amount consumed by the Borlotti bean buyers. Lopez et al. (2013) studied the effect of cooking on the phenolic composition of dark common beans (*P. vulgaris*) [20]. After boiling, the anthocyanin content decreased by 68% and flavanones decreased by 53%. The most abundant flavanols found were quercetin and kaempferol, that decreased by 44% and 5.82% upon cooking, respectively. Of the hydroxycinnamic acids identified, ferulic acid decreased by 48%, sinapic acid by 56% and p-coumaric acid by 60%. During soaking, seed coat polyphenols such as flavonoids and hydroxycinnamic acids are solubilized and diffused into the soaking water, contributing to polyphenol decrease [46,47].

In the present study, cooking water presented higher TPC values than raw bean methanolic extract (Figure 1). A possible explanation for this behaviour is that thermal processing might have stimulated the release of bound polyphenols more efficiently than acidified methanol. Chen et al. (2015) found that thermal processing stimulated the release of bound polyphenols (flavonols and hydroxycinnamic acids) [48]. Similarly, Ranilla et al. (2009) observed that thermal treatment of common beans (*P. vulgaris*) increased the amount of free phenolic acids and flavonols measured [46]. Cooking treatments soften the cell walls of vegetable food, enhancing the extraction of polyphenols [49]. Our results suggest that cooking processing releases higher amounts of polyphenols than solvent extraction. In the present study, enzymatic digestion released a twenty-fold higher polyphenol release compared to methanolic polyphenol extraction. This implies that the solvent extraction commonly used to extract polyphenols may underestimate the polyphenol content of beans.

The most significant finding of this work was that α-amylase hydrolysis was responsible for the release of around 76% of the polyphenols liberated upon in vitro digestion. This provides strong evidence that polyphenols interact with starch structures in nature, and therefore starch can be considered as the main limiting factor for polyphenol bioaccessibility in Borlotti beans, which has not been reported before. Fluorescence microscopy of isolated starch granules revealed strong fluorescence within the granules, providing further evidence that polyphenols are embedded within the starch granule structure and are liberated from the starch matrix during digestion. The inclusion of polyphenols into the starch granule may occur during granule formation. Legume starch has been shown to have a C-type polymorphic arrangement within starch granules, which in effect is characterized by the presence of both A- and B-type polymorphs within the same granule [47]. We postulate that polyphenols may have an effect on the polymorphic conformation of legume starch, potentially by facilitating a B-type configuration where the space between chains is occupied by polyphenol molecules. Further studies are required to understand the nature of polyphenol interaction within native starch granules. Polyphenols have been shown to be weak inhibitors of amylase and slow down starch digestion [48,49,50] and the complexation of polyphenols with starch has been shown to reduce the post-prandial glucose response in mice [27]. Other studies have shown no effect [51]. It is therefore possible that naturally occurring polyphenol–starch complexes in beans contribute to their low glycemic potential [52], but further research is required to elucidate structure/function relationships.

The mechanisms by which polyphenols interact with starch are not yet clear. Examples of non-covalent interactions between flavonoids and starch have been proposed [24,53]. Wu et al. (2009) observed that the addition of flavan-3-ols from tea to rice starch retards retrogradation by preventing re-crystallization of amylopectin, although the mechanism of this is not fully understood [25]. Barros et al. (2012) observed that proanthocyanidins interacted better with amylose than with amylopectin while monomeric polyphenols were shown to interact equally with both amylose and amylopectin [26]. Chai et al. (2013) demonstrated that self-assembled complexes between tea polyphenols and amylose were formed by hydrogen bonding [27]. Small molecules may interact with amylose by forming inclusion complexes with single left-handed helices, known as V-type inclusion complexes [54]. The formation of a V-type complex has been reported for genistein and corn starch [28,29]. This interaction has not yet been studied for other polyphenols or in native starch systems.

The digestion protocol used in this study was optimized to achieve the maximum possible digestion of starch (Figures S1 and S2). The pancreatic α-amylase concentration is similar to the proposed used in the harmonized INFOGEST in vitro digestion method [39,40], however, our digestion time is much longer (16 h, compared to 2 h). It should be noted that the INFOGEST method was developed primarily to study protein and lipid digestion, and has not been optimized to study starch digestion. Furthermore, there is no harmonized protocol to study bioaccessibility. Angelino et al. (2017) highlighted that there is considerable variability between in vitro studies, mainly resulting from the use of different methodologies, highlighting the need for standardization [55]. According to Fernandez-Garcia et al. (2019) bioaccessibility is defined as ‘the quantity of a specific compound that is released from the food matrix during digestion and is available to the intestinal cells for potential absorption’ [38]. Meanwhile, Ribas-Agusti et al. (2018) define it as ‘the fraction of compounds that is released from the food matrix during digestion and becomes available for small intestinal absorption’ [56]. Shahidi and Peng define bioaccessibility using a rather more physiological/pharmacological approach as ‘the digestion and absorption efficiency (or digestibility and absorptivity) of a certain food constituent or drug ingested by oral administration, normally expressed as a percentage of the actual amount released and absorbed constituent to its total content’ [57]. We have used the definition by Fernandez-Garcia et al. (2019). There is a lack of knowledge of bean starch digestion and polyphenols bioaccessibility in vivo. Further work is required to understand the physiological relevance of our observations.

Around 16% of the TPC released upon in vitro digestion were liberated after the protease hydrolysis of Borlotti beans. Polyphenols may bind with proteins mainly by non-covalent interactions (hydrophobic, van der Waals, hydrogen bridge binding and ionic interactions) [24]. In this study, the Folin–Ciocalteu method was used to determine the number of polyphenols released upon in vitro digestion. This commonly used method is based on the electron transfer reactions that take place between polyphenols and the Folin–Ciocalteu reagent [58]. It has been reported that compounds like peptides may also react with the Folin–Ciocalteu reagent [59]. Peptides have indeed been identified in abundance following the pepsin–pancreatin hydrolysis of the Negro Jamapa bean (*P. vulgaris*) protein [60]. Therefore, it can be assumed that digestion supernatants of Borlotti beans may also contain similar bioactive peptides, affecting the TPC results.

In this study, a further polyphenol extraction was achieved after acid hydrolysis of Borlotti bean fibre. It is known that polyphenols bind with fibre components such as cellulose, hemicellulose (e.g., arabinoxylans), lignin and pectin, and are normally present as glycosides, linked through a hydroxyl group with single or multiple sugars (*O*-glycosides) or through carbon–carbon bonds (*C*-glycosides) [61]. Under physiological conditions, these polyphenols would only become bioavailable following metabolism by the microbiota [62].

As shown by LC–MS results, the cooked Borlotti bean extract contained a mixture of polyphenols including catechin, apigenin and hydroxycinnamic acid derivatives, which are likely responsible for the anti-inflammatory effects observed in LPS-stimulated RAW264.7 macrophages. In a previous study, chlorogenic acid inhibited LPS-induced RAW264.7 macrophages [63]. Kim et al. (1999) found that apigenin and other flavonoids (wogonin, luteolin, tectorigenin, and quercetin) inhibited NO production in LPS-stimulated RAW 264.7 cells [64]. Comalada et al. (2006) found that low concentrations (25 and 50 mM) of apigenin reduces the NF–kB regulated cytokines iNOS and TNFα, as well as NO values in bone-marrow-derived mouse macrophages [65].

Studies using extracts from common bean (*P. vulgaris*) cultivars have reported anti-inflammatory effects in vitro and in vivo. Garcia-Lafuente et al. (2014) found that round purple bean extracts reduced NO production and reduced IL-1b and IL-6 mRNA expression of LPS-stimulated macrophages [66]. Chen et al. (2017) found that Borlotti bean acetone extracts strongly attenuated the secretion of IL-8 in human colonic carcinoma Caco-2 cells [67]. In vivo, Zhang et al. (2014) found that bean-rich diets reduced local and systemic inflammation in a mouse model of acute colitis, but also increased mucosal damage and inflammation [68].

The concentrations of Borlotti bean extract are biologically relevant. According to Scalbert and Williamson (2000) the maximum concentrations of polyphenols in the blood stream after the consumption of polyphenol-rich foods are about 0.1–1 mM [69]. The TPC values of cooked Borlotti bean extract were 1.27 mg GAE/g of sample. If this value is converted to mM (calculations considering mM of Gallic acid), the values tested (50–200 mg of sample/mL) would range from 0.37–1.5 mM, making the concentrations tested biologically relevant. Overall, the evidence supports the hypothesis that bean polyphenols have anti-inflammatory activity through the inhibition of NF–κB and downstream pathways, but more research is needed to understand whether lower concentrations would be more effective, and the effect of mixtures of polyphenols, bean extracts and bean-rich diets on other inflammatory pathways, in vitro and in vivo. Furthermore, more target genes and more cell lines could be used for future studies in order to have a better understanding of bean extract on other tissues and other biological pathways. Beans extracts contain a mixture, and test individual compounds.

The present study presents interesting data regarding possible associations between polyphenols and bean starch, which have not been reported previously. Also, cooked Borlotti bean extract exerted a moderate anti-inflammatory effect indicating the possible health benefits of this legume. However, we consider that this study has a few limitations that should be addressed in future work, including: applying physiologically relevant digestion conditions, assessing bioavailability in vivo, testing the effect of lower concentrations of extract and single/combinations of compounds on NF–κB and downstream pathways.

## 5. Conclusions

Our data provide new evidence that digestion, in particular starch enzymatic hydrolysis, may increase polyphenol bioaccessibility. It is currently unknown whether polyphenol/starch interactions are also naturally present in other beans and other plant foods. Further studies are required to understand the mechanism of starch polyphenol interactions, how these are formed in planta and how they are disrupted during food processing. Furthermore, the physiological relevance of these interactions is not yet clear. Further research, in vitro and in vivo, is needed to understand these interactions and their potential impact on human metabolism and health, and the implications of the interactions on polyphenol bioactivity. Furthermore, studies are needed to better understand the effect of individual polyphenols or combinations, as well as polyphenol-rich diets, on anti-inflammatory pathways and disease prevention.

## Figures and Tables

**Table 1:** Primer sequence information of target genes.

Target	Forward Sequence (5′-3′)	Reverse Sequence (5′-3′)
*β-actin*	CCTCTATGCCAACACAGTGC	CCTGCTTGCTGATCCACATC
*IL-6*	AGTTGCCTTCTTGGGACTGA	CAGAATTGCCATTGCACAAC
*IL-1b*	CAGGCAGGCAGTATCACTCA	AGCTCATATGGGTCCGACAG
*iNOS*	GCAGCCTGTGAGACCTTTG	GCATTGGAAGTGAAGCGTTTC

**Table 2:** HPLC-MS analysis of cooked Borlotti bean extract.

Peak	Retention Time (min)	Main Ions Detected	Possible Parent Compound
1	0.4	+291.31 −289.27 +579.42	Procyanidin B-type
2	0.5	−648.99	apigenin-7-O-(6″-malonyl-apiosyl-glycoside)
3	0.5	+355.82	chlorogenic acid derivative
4	0.5	+959.33 −958.37	not identified
5	0.6	+355.82 −515.20	chlorogenic acid glycoside
6	0.6	+379.41 −355.45	not identified
7	0.7	+355.41 −227.2	not identified
8	1.0	+338.62 −521.12	petunidin-3-O-(6′′-acetyl glycoside)
9	1.1	+338.58 −521.17	petunidin-3-O-(6′′-acetyl-glycoside)
10	1.1	+338.63 −521.18	petunidin-3-O-(6′′-acetyl-glycoside)

## Supplementary Materials

The following are available online at https://www.mdpi.com/2072-6643/12/2/295/s1. Figure S1. Reducing sugars determined by the 3,5-dinitrosalicyclic acid (DNS) assay released upon Borlotti bean digestion with pancreatic α-amylase (PA). (a) effect of different concentrations of PA at three concentrations of enzyme (10, 50 and 100 U/mL) at different incubation times (2, 4, 6 and 16 h); (b) effect of PA digestion with or without subsequential digestion with 80 U/mL amyloglucosidase (AMG) digestion at 2 h and 16 h. A greater starch hydrolysis was observed when cooked Borlotti bean was digested with PA for 16h and a subsequential digestion with AMG. Figure S2. Reducing sugars determined by the DNS assay (a) and HPAEC-PAD (b) present in supernatants of Borlotti bean digestion with different amounts of PA (100–1000 U/mL) for 16 h and a posterior hydrolysis with of AMG (80 U/g). Figure S3. LC-MS chromatograms of cooked Borlotti bean extract.
