# Supplementary material for: Starch Digestion Enhances Bioaccessibility of Anti-Inflammatory Polyphenols from Borlotti Beans (Phaseolus vulgaris)
Source: Nutrients. 2020 Jan 22;12(2):295. doi: 10.3390/nu12020295 (PMC7070432; doi:10.3390/nu12020295)
Supplement: Supplementary file 1 [file nutrients-12-00295-s001.pdf]

Supplementary material

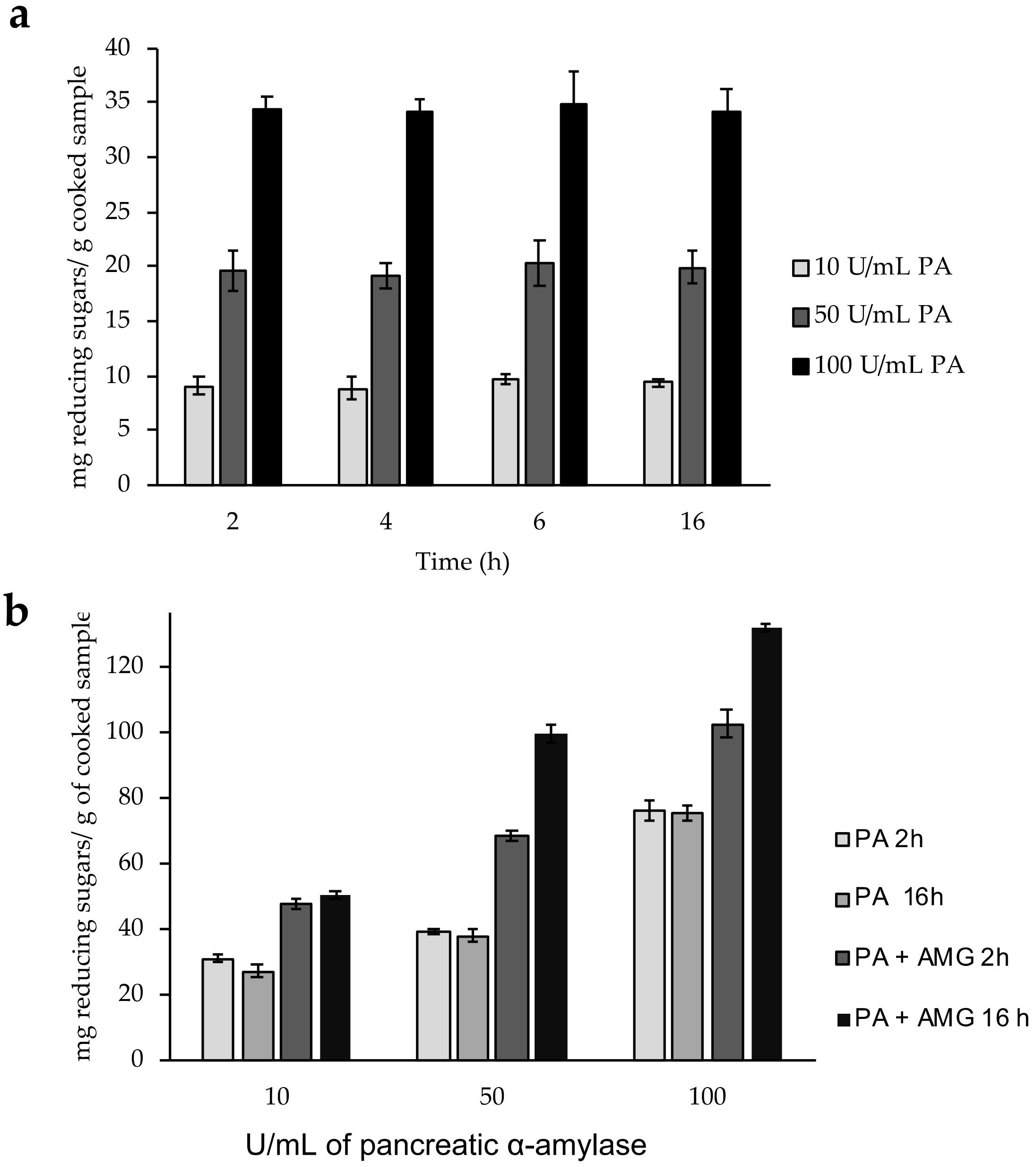

Figure S1. Reducing sugars determined by the 3,5- dinitrosalicyclic acid (DNS) assay released upon Borlotti bean digestion with pancreatic α-amylase (PA). a) effect of different concentrations of PA at three concentrations of enzyme (10, 50 and 100 U/mL) at different incubation times (2, 4, 6 and 16 h); b) effect of PA digestion with or without subsequential digestion with 80 U/ml amyloglucosidase (AMG) digestion at 2 h and 16 h. A greater starch hydrolysis was observed when cooked borlotti bean was digested with PA for 16h and a subsequential digestion with AMG.

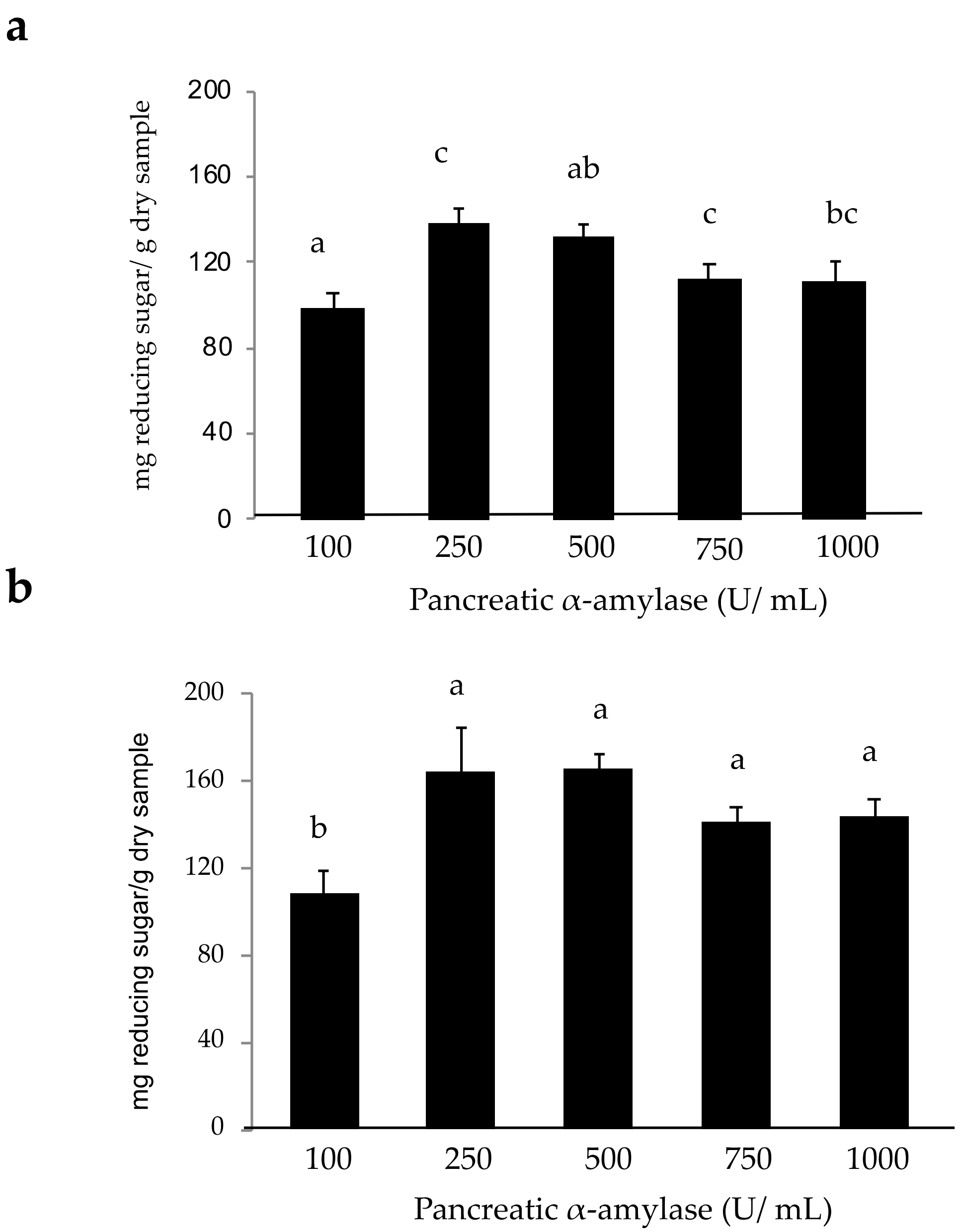

Figure S2. Reducing sugars determined by the DNS assay (a) and HPAEC-PAD (b) present in supernatants of Borlotti bean digestion with different amounts of PA (100 – 1000 U/ml) for 16 h and a posterior hydrolysis with of AMG (80 U/g).

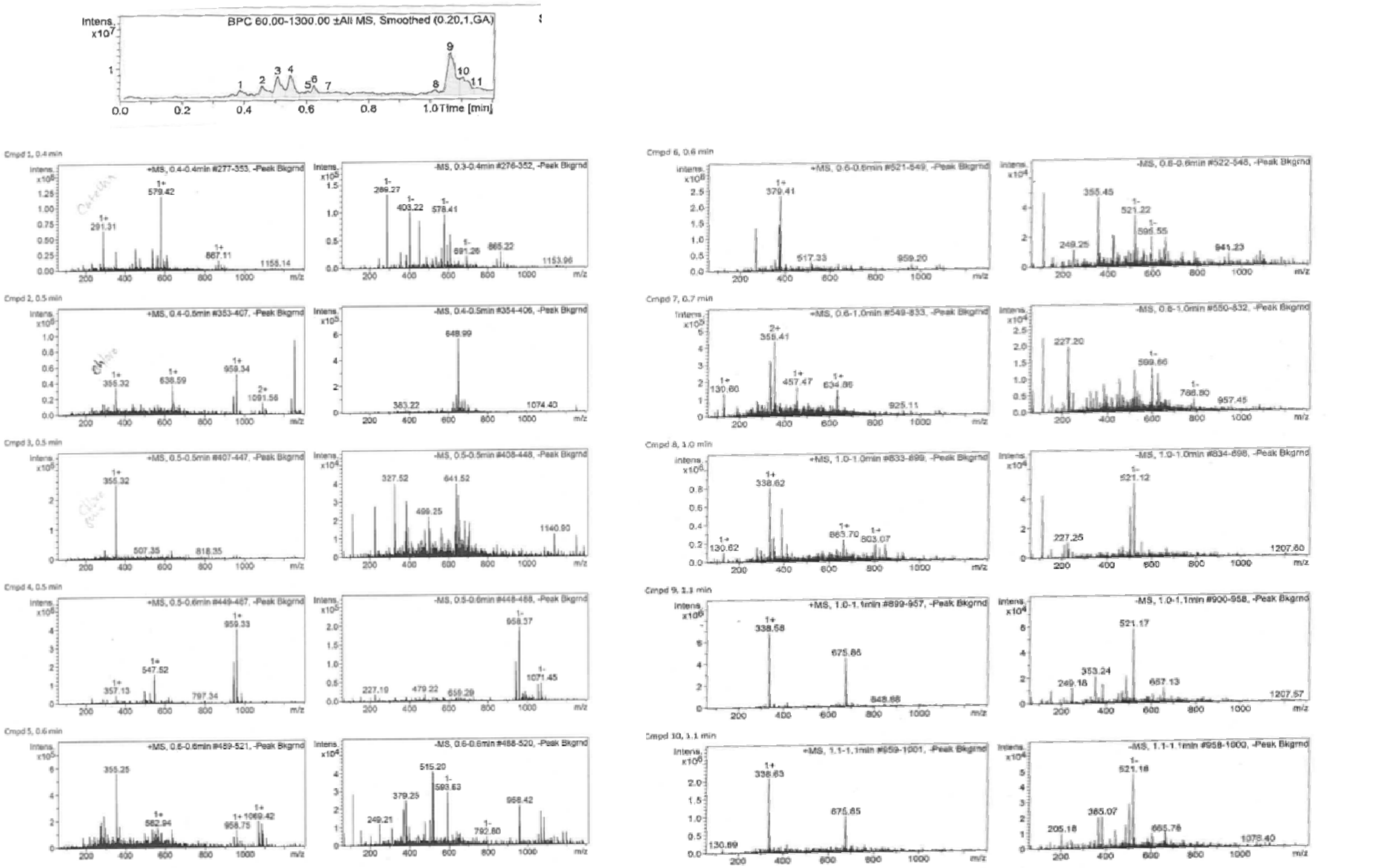

Figure S3. LCMS chromatograms of Borlotti bean extract analysis.
